# Experimental application of an automated alignment correction algorithm for geological CT imaging: phantom study and application to sediment cores from cold-water coral mounds

**DOI:** 10.1186/s41747-019-0091-8

**Published:** 2019-03-12

**Authors:** Stephan Skornitzke, Jacek Raddatz, André Bahr, Gregor Pahn, Hans-Ulrich Kauczor, Wolfram Stiller

**Affiliations:** 1 Diagnostic and Interventional Radiology (DIR), Heidelberg University Hospital, Im Neuenheimer Feld 110, 69120 Heidelberg, Germany; 2 Institut für Geowissenschaften, J. W. Goethe-Universität, Frankfurt am Main, Germany; 3 Institute of Earth Sciences, Ruprechts-Karls-Universität Heidelberg, Heidelberg, Germany

**Keywords:** Alignment correction, Cold-water corals, Phantoms (imaging), Sediment core, Tomography (x-ray computed)

## Abstract

**Abstract:**

**Background:**

In computed tomography (CT) quality assurance, alignment of image quality phantoms is crucial for quantitative and reproducible evaluation and may be improved by alignment correction. Our goal was to develop an alignment correction algorithm to facilitate geological sampling of sediment cores taken from a cold-water coral mount.

**Methods:**

An alignment correction algorithm was developed and tested with a CT acquisition at 120 kVp and 150 mAs of an image quality phantom. Random translation (maximum 15 mm) and rotation (maximum 2.86°) were applied and ground-truth was compared to parameters determined by alignment correction. Furthermore, mean densities were evaluated in four regions of interest (ROIs) placed in the phantom low-contrast section, comparing values before and after correction to ground truth. This process was repeated 1000 times. After validation, alignment correction was applied to CT acquisitions (140 kVp, 570 mAs) of sediment core sections up to 1 m in length, and sagittal reconstructions were calculated for sampling planning.

**Results:**

In the phantom, average absolute differences between applied and detected parameters after alignment correction were 0.01 ± 0.06 mm (mean ± standard deviation) along the *x*-axis, 0.11 ± 0.08 mm along the *y*-axis, 0.15 ± 0.07° around the *x*-axis, and 0.02 ± 0.02° around the *y*-axis, respectively. For ROI analysis, differences in densities were 63.12 ± 30.57, 31.38 ± 32.10, 18.27 ± 35.57, and 9.59 ± 26.37 HU before alignment correction and 1.22 ± 1.40, 0.76 ± 0.9, 0.45 ± 0.86, and 0.36 ± 0.48 HU after alignment correction, respectively. For sediment core segments, average absolute detected parameters were 3.93 ± 2.89 mm, 7.21 ± 2.37 mm, 0.37 ± 0.33°, and 0.21 ± 0.22°, respectively.

**Conclusions:**

The alignment correction algorithm was successfully evaluated in the phantom and allowed a correct alignment of sediment core segments, thus aiding in sampling planning. Application to other tasks, like image quality analysis, seems possible.

## Key points

- Simulated misalignment in an image quality phantom could be corrected
- Average misalignment after correction was below a single voxel in phantom
- CT imaging facilitates sampling process of corals from sediment cores
- Alignment correction can improve accuracy of geological sampling planning

## Background

Geometrically accurate positioning of scanned objects as well as of patients is important for ensuring adequate image quality and stability of measured computed tomography (CT) numbers. Furthermore, in order to minimise patient radiation exposure, patients should be centred in the field of view of the CT system. Even more so, quantitative evaluations, *e.g.*, of standardised image quality phantoms, rely on the exact positioning of the imaged object to produce reproducible and accurate results [1].

CT imaging becomes increasingly important in geosciences, especially in sedimentological cold-water coral (CWC) studies [2, 3]. High-resolution CT data not only provides information about qualitative occurrences of CWC fragments, but also enables the quantification of visual observations of CWC skeletons in sediment cores from clast-size and clast-orientation analyses and helps to further understand CWC mound growth, combined with radiometric age determinations as radiocarbon or ^230^Th/U [2]. The qualitative information on coral occurrences in the sediment core further helps for accurate sampling with minimal disturbance of sediment and other fragments by allowing to locate CWC fragments below the surface.

Sampling of sediment cores is usually carried out with a resolution of up to 1 cm. Coral clast sizes of about 2 cm may easily disturb sediment during sampling; therefore, non-destructive information from CT imaging can greatly improve the sampling process. Based on the coral clast sizes, the accurate orientation of the CT image data used for visualisation needs to be better than 0.5 cm. As discussed above, geometrically exact object positioning is important for quantitative evaluations based on CT image data and misalignment of cores may lead to deviations in the expected and actual location of coral clasts within the sediment cores.

Therefore, the main goal of this study was the development of an alignment correction algorithm to facilitate the CT image-based analysis and systematic sampling of sediment cores. A simple alignment correction algorithm was developed for restoring alignment of the imaged cores with the main axes of the scanner system and evaluated using image data of a standard image quality phantom. The algorithm was subsequently used to provide accurate sagittal reconstructions for the planning of core sampling.

## Methods

### Sediment cores

**Fig. 1:**
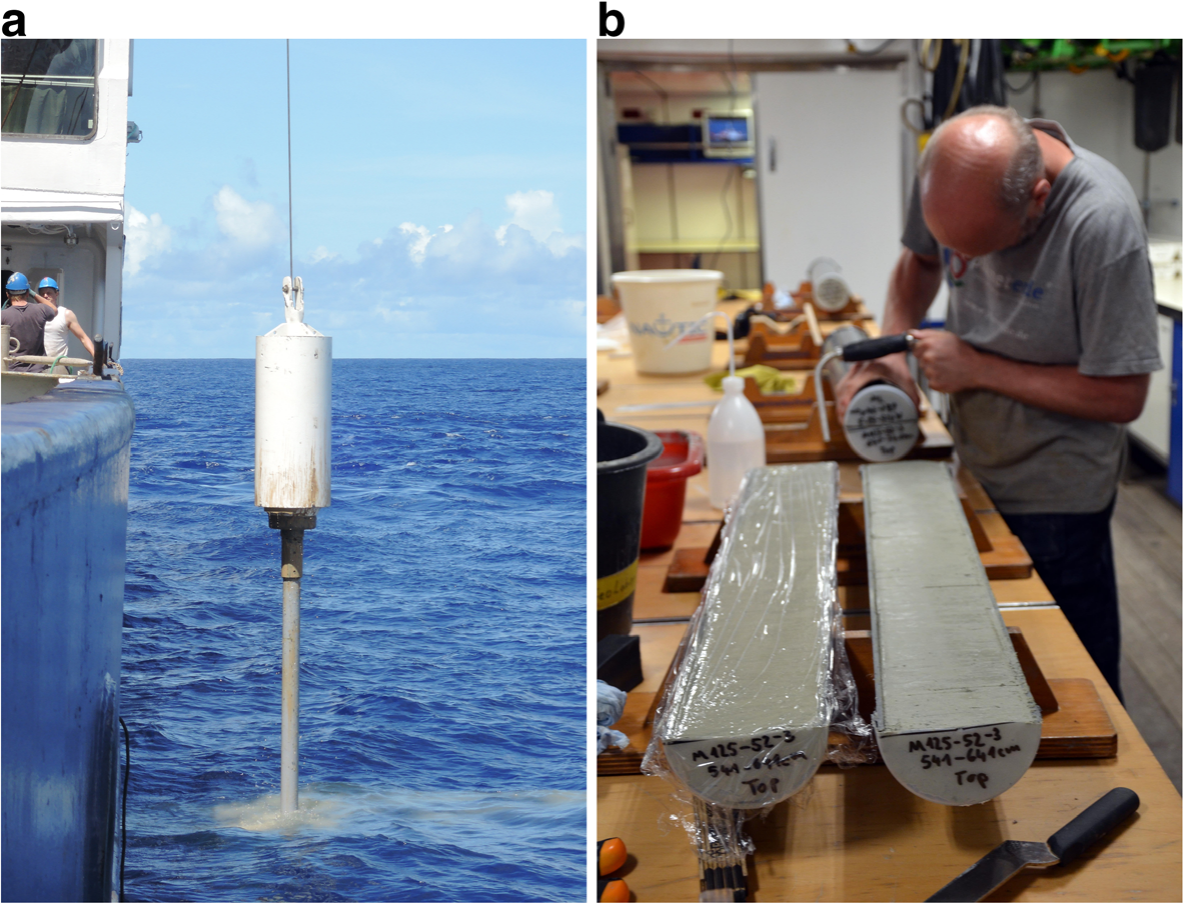
**a** Retrieval of sediment cores off the shore of Brazil during the research cruise M125. **b** Example of a sediment core equivalent to those used in this study, cut in half for sampling of core contents. Samples are only taken from one half of the core, while the other half is archived for reference (photo courtesy of J. Hoffmann)

During the recently performed research cruise M125 with the German research vessel *Meteor*, a 5.83-m-long sediment core was retrieved from a 25-m-high CWC mound in 860-m water depth off the coast of Brazil (station M125–34-2; 21°56,959′ S, 39°32,031′ W; Fig. 1) [4]. This CWC-bearing sediment core has the potential to provide an insight into the origin, growth, and demise of these mounds as well as to carry out paleoceanographic reconstructions [5, 6].

**Fig. 2:**
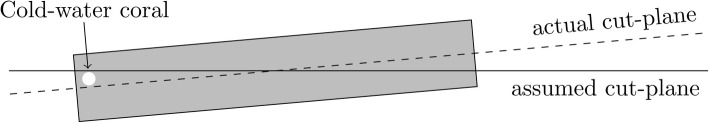
Schematic example of the effect of core misalignment on core sampling planning (coronal plane, not to scale). When calculating sagittal reconstructions, misalignment might lead to a misrepresentation of sample locations (see also Fig. 6). In consequence, samples might be missed and other samples might be destroyed during the sampling process

On board of the vessel, the sediment core was split into sections of 1 m, but remained unopened as a prerequisite for subsequent CT imaging at the Clinic of Diagnostic and Interventional Radiology (DIR) of Heidelberg University Hospital, Heidelberg, Germany. For paleoceanographic analysis, the 1-m sections are cut by a diamond saw (blade thickness 2–3 mm) along the longitudinal axes while still frozen in order to avoid sediment and coral disturbances (see Fig. 1). As described in detail below, CT acquisitions of the sediment core sections containing CWCs were performed for sampling planning. However, despite taking great care to align cores with the main axes of the CT scanner system using the system’s laser sight, shape, length, and mechanical deformation of cores may influence accurate core alignment, thus leading to deviations in expected and actual location of coral clasts within the sediment cores, as detailed in Fig. 2. To allow for the calculation of geometrically accurate sagittal reconstructions of CT acquisitions of sediment core sections containing CWCs, an alignment correction algorithm was developed for restoring alignment of imaged cores with the main axes of the CT scanner’s coordinate system. The developed algorithm was evaluated for its performance using CT acquisitions of a standard image quality phantom.

### Image acquisition

#### Phantom

For evaluating the algorithm performance, a CT acquisition of a standard image quality phantom was used (ConeBeam Phantom, QRM Quality Assurance in Radiology and Medicine GmbH, Möhrendorf, Germany). The phantom was scanned at 120 kVp tube potential and 150 mAs tube current-time product with a pitch of 0.95 (SOMATOM Definition Flash; Siemens Healthineers, Forchheim, Germany). Images were reconstructed with a soft-tissue kernel (B30f), a slice thickness/increment of 1 mm/1 mm, and an isotropic voxel size of 0.39 mm by 0.39 mm.

#### Sediment cores

Six sediment core sections from cold-water coral mounds taken during research cruise M125, as described above, were imaged using the abovementioned CT scanner system (SOMATOM Definition Flash). The cores consist of sediment embedded in the core liner. Individual core sections had a length of up to 1 m and a diameter of approximately 12 cm. For image acquisition, core sections were manually aligned with the main axes of the CT scanner coordinate system using the integrated laser sight system. Image acquisitions were performed at 140 kVp tube potential and 570 mAs tube current-time product with a pitch of 0.4. Images were reconstructed with iterative reconstruction (ADMIRE, Siemens Healthineers) using a sharp kernel (I70h level 3), a slice thickness/increment of 0.5 mm/0.3 mm, and an isotropic voxel size of 0.35 mm by 0.35 mm.

### Alignment correction algorithm

**Fig. 3:**
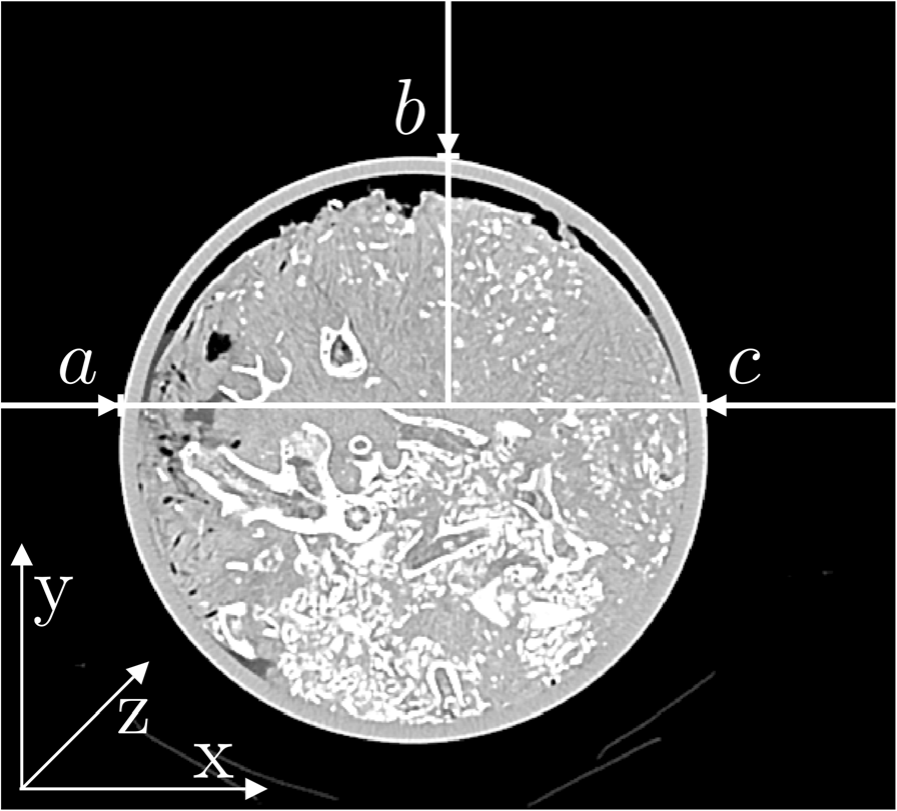
Image example of an axial CT acquisition of a sediment core, showing the coordinate system referred to as the *CT scanner coordinate system* in the manuscript and illustrating the line search to determine size and position of the scanned object on axial images. Three points **a**, **b**, and **c** are determined by a bisection algorithm along the search direction. Note the distribution of coral fragments in the core and the gap between sediment and core liner at the top of the image

A previously developed algorithm for positioning of regions of interest (ROIs) in image quality phantoms was adapted for the task of correcting sediment core alignment [7]. The algorithm was designed to restore alignment of a scanned cylindrical object with the longitudinal axis of the scanner as defined by the axes of the image stack (Fig. 3). The algorithm performs the following steps:

1. Detecting three points on the boundary of the cylinder by one-dimensional edge detection on each individual image
2. Calculating circle equation for the detected object on each individual image, determining centre position and radius
3. Dividing image stack into segments based on changes in centre position and radius
4. Determining orientation of the longest segment based on centre positions relative to the CT coordinate system
5. Applying alignment correction based on determined orientation by translating and rotating images in the inverse direction

First, three points on the boundary of the cylindrical object are determined by one-dimensional edge detection for each image of the image stack. Edge detection is performed twice along the *x*-axis of the reconstructed images from the outside to the inside of the field of view (once in each direction). The third line search is performed along the *y*-axis, from top-to-bottom, thus omitting the CT scanner’s patient table (see Fig. 3). The line search is performed along the central rows of the image, using a bisection algorithm that minimises the variance of grey values of the two sections [7].

Second, using the three detected points on each image, the centre coordinates and radius, *x*_0_, *y*_0_, and *r*, defined by the circle equation, are determined for each image:

1 $$ {\left(x-{x}_0\right)}^2+{\left(y-{y}_0\right)}^2={r}^2 $$

**Fig. 4:**
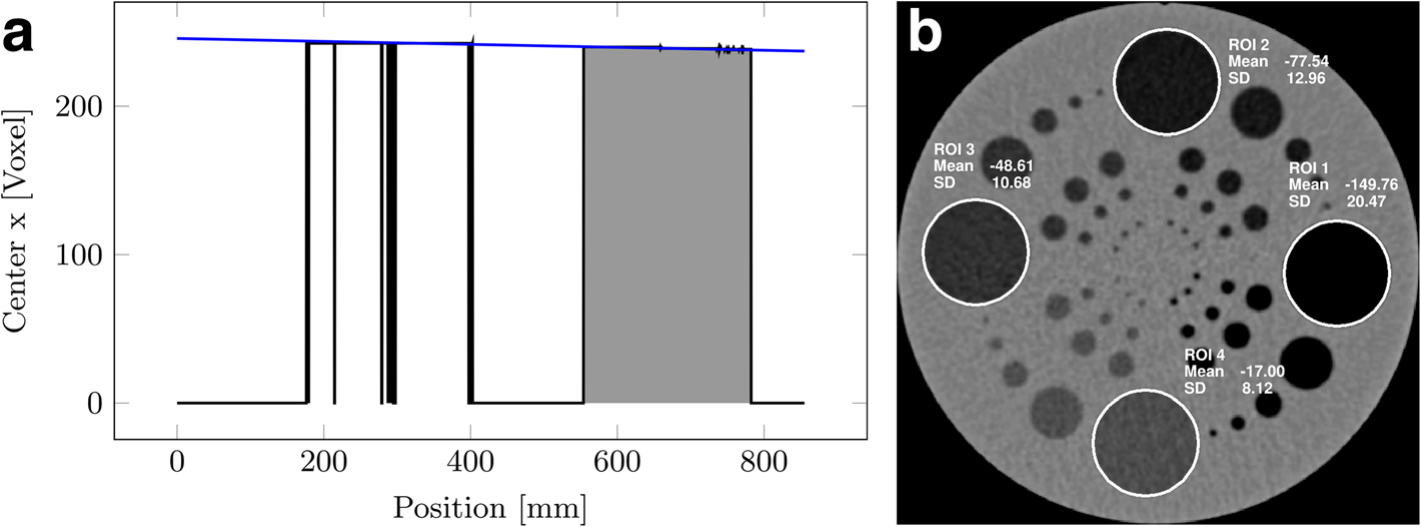
**a** Example of a plot of detected *x*-coordinates of the object centre for valid segments (black line), plotted over slice position, for a sediment core. The blue line indicating core position in *x* was fit to the largest segment (grey area). **b** Example axial CT image of the phantom used for evaluation of the algorithm, showing the placement of regions of interest (ROIs) in the low-contrast section of the phantom

Third, image segments are defined based on the changes in centre position and radius between images. Here, a segment is a continuous number of images, which have a very similar centre and radius: Images belong to the same segment, if the relative change in radius between two images is below 0.5% and if the change in position is below two voxels in *x*- and *y*-direction. Segments shorter than five images are removed. Ideally, this step will yield one segment containing the entire core (Fig. 4).

Fourth, the longest segment is chosen as the one most likely belonging to a major section of the object. A straight line is fit by regression through the determined circle centres of the chosen segment (see Fig. 4). Based on the line equation, the offset relative to the centre of the reconstructed image stack (and thus the CT coordinate system) and the rotation around *x*- and *y*-axis can be determined.

Fifth, the image is translated and rotated using the inverse of the determined offset and rotation, using the “Insight Segmentation and Registration Toolkit” (ITK, Kitware Inc., Clifton Park, NY, USA) [8].

### Phantom evaluation

For evaluating algorithm performance, the CT acquisition of a standard image quality phantom was randomly rotated and translated, alignment correction was performed, and the algorithmically determined misalignment was compared to the ground truth.

Parameters for rotation and translation were randomly determined according to a uniform distribution. The maximum translation along *x*- and *y*-axis was up to 15 mm each. The maximum rotation around *x*- and *y*-axis was up to 2.86° each (corresponding to 0.05 rad). For larger translations and rotations, parts of the phantom may be outside of the reconstructed field of view, making a successful alignment correction impossible. Rotations and translations were applied using the abovementioned tool. For evaluation, the process was repeated 1000 times, algorithmically determined rotation and translation were compared to the ground truth of the generated parameters, and descriptive statistics were calculated.

Furthermore, four ROIs were placed in the low-contrast section of the phantom and mean CT numbers of the ROIs were compared for the initial acquisition, before and after alignment correction (see Fig. 4).

### Statistical analysis

Statistical testing was performed using SAS 9.4 (SAS Institute, Cary, NC, USA). Differences between detected parameters and ground truth and differences in measured CT numbers in ROIs were analysed using a one-sample two-tailed Student *t* test. The null hypothesis was that mean differences were equal to zero, which was rejected at a significance level of *α* = 0.05. Normality of data was assessed visually, using histograms and Q-Q scatterplots.

### Application to sediment cores

Images of sediment core sections were corrected for their alignment using the algorithm described above. Detected misalignment, given as translation and rotation, was recorded. After alignment correction, sagittal reconstructions were calculated, showing images in parallel to the plane where the core is cut for sampling, thus allowing for planning of core sampling [7]. Sagittal images were reconstructed at a slice thickness of 5 mm with an increment of 5 mm, adding a digital ruler as an overlay to the image. Using the sagittal images, corresponding to a different depth below the surface, CWC fragments were identified and located, aiming to remove the samples from the sediment core with minimal destructive effect on neighbouring CWC fragments and surrounding sediment.

## Results

### Phantom study

Average differences between generated parameters and the parameters detected by the algorithm, which can be used to investigate a bias in the difference, were -0.03 ± 0.09 mm for translation along the *x*-axis, 0.00 ± 0.16 mm for translation along the *y*-axis, 0.11 ± 0.11° for rotation around the *x*-axis, and 0.00 ± 0.05° for rotation around the y-axis. Mean differences differ significantly from 0, except for the difference in the translation along the *y*-axis (Table 1).

**Table 1:** Reported *p* values from the one-sample Student *t* test, comparing determined parameters and measured mean CT numbers to ground truth

Alignment parameter	*p* value
Translation along *x*	0.000*
Translation along *y*	0.952
Rotation around *x*	0.000*
Rotation around *y*	0.000*
ROI measurement	
ROI 1–4	0.000*

*ROI* region of interest

*Reported as *p* < 0.0001 by the evaluation software and thus rounded to 0.000

**Fig. 5:**
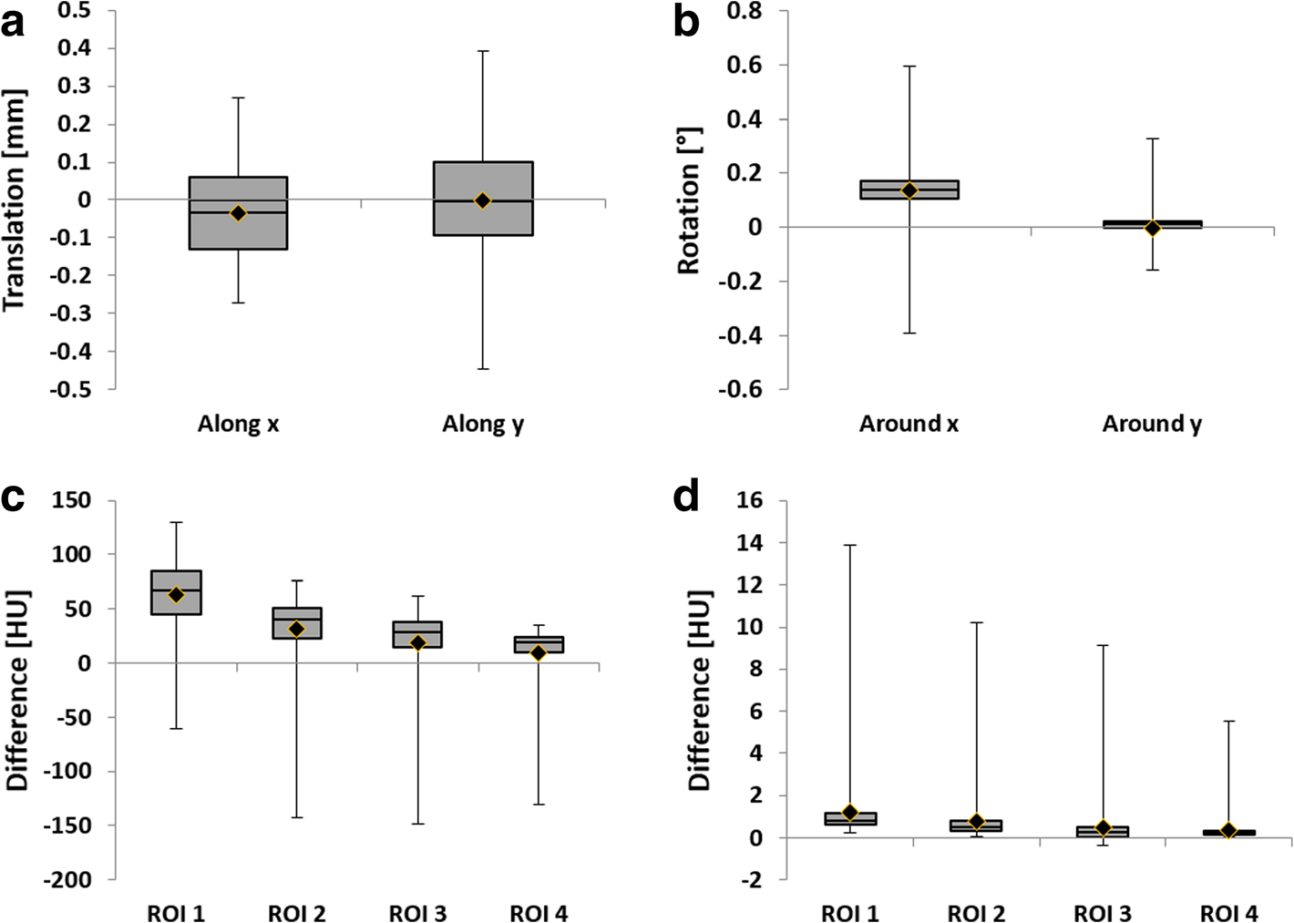
Results of the phantom evaluation of algorithmically determined parameters versus ground truth. Boxes indicate upper and lower quartile and median, diamonds indicate mean, and maximum and minimum are indicated by whiskers. **a** Difference in translation. **b** Difference in rotation. **c** Difference in CT numbers measured in regions of interest (ROIs) before correction. **d** Difference in CT numbers measured in ROIs after correction. Note the different quantities and units and the different scaling of the *y*-axes

Average absolute differences between generated parameters and the parameters detected by the algorithm, which can be used to determine the magnitude of the difference, were 0.01 ± 0.06 mm for translation along the *x*-axis, 0.11 ± 0.08 mm for translation along the *y*-axis, 0.15 ± 0.07° for rotation around the *x*-axis, and 0.02 ± 0.02° for rotation around the *y*-axis (Fig. 5).

For ROI analysis, mean CT numbers measured in the original dataset were -149.76 HU, -77.54 HU, -48.61 HU, and -17.00 HU. Averages of ROI measurements before alignment correction were -86.64 ± 30.57 HU, -46.16 ± 32.10 HU, -30.34 ± 35.57 HU, and -7.41 ± 26.37 HU and -148.54± 1.40 HU, -76.78 ± 0.90 HU, -48.16 ± 0.86 HU, and -16.64 ± 0.48 HU after alignment correction, respectively. Compared to the original dataset, the average difference before alignment correction was 63.12 ± 30.57 HU, 31.38 ± 32.10 HU, 18.27 ± 35.57 HU, and 9.59 ± 26.37 HU, while the average difference after alignment correction was 1.22 ± 1.40 HU, 0.76 ± 0.9 HU, 0.45 ± 0.86 HU, and 0.36 ± 0.48 HU, respectively (see Fig. 5). Differences in measured CT numbers between ground truth and alignment corrected data were statistically significant (see Table 1).

### Sediment cores

Core alignment could be corrected for all six sediment cores. Average detected parameters, showing a potential bias in misalignment, were -0.76 ± 4.82 mm for translation along the *x*-axis, -7.21 ± 2.37 mm for translation along the *y*-axis, 0.37 ± 0.33° for rotation around the *x*-axis, and 0.06 ± 0.30° for rotation around the *y*-axis (Table 2).

**Table 2:** Misalignment algorithmically determined for CT acquisitions of sediment cores

	Translation along *x* [mm]	Translation along *y* [mm]	Rotation around *x* [°]	Rotation around *y* [°]
Core 1	-4.92	-4.92	0.05	-0.19
Core 2	-0.35	-7.73	0.31	-0.10
Core 3	-2.46	-8.79	0.54	-0.08
Core 4	8.44	-9.84	1.01	-0.07
Core 5	1.05	-8.79	0.24	0.12
Core 6	-6.33	-3.16	0.09	0.70

Average absolute parameters, showing the magnitude of the misalignment, were 3.93 ± 2.89 mm for translation along the *x*-axis, 7.21 ± 2.37 mm for translation along the *y*-axis, 0.37 ± 0.33° for rotation around the *x*-axis, and 0.21 ± 0.22° for rotation around the *y*-axis. In comparison to average, non-absolute results, translation along the *y*-axis was negative for all cores, while rotation around the *x*-axis was positive for all cores.

**Fig. 6:**
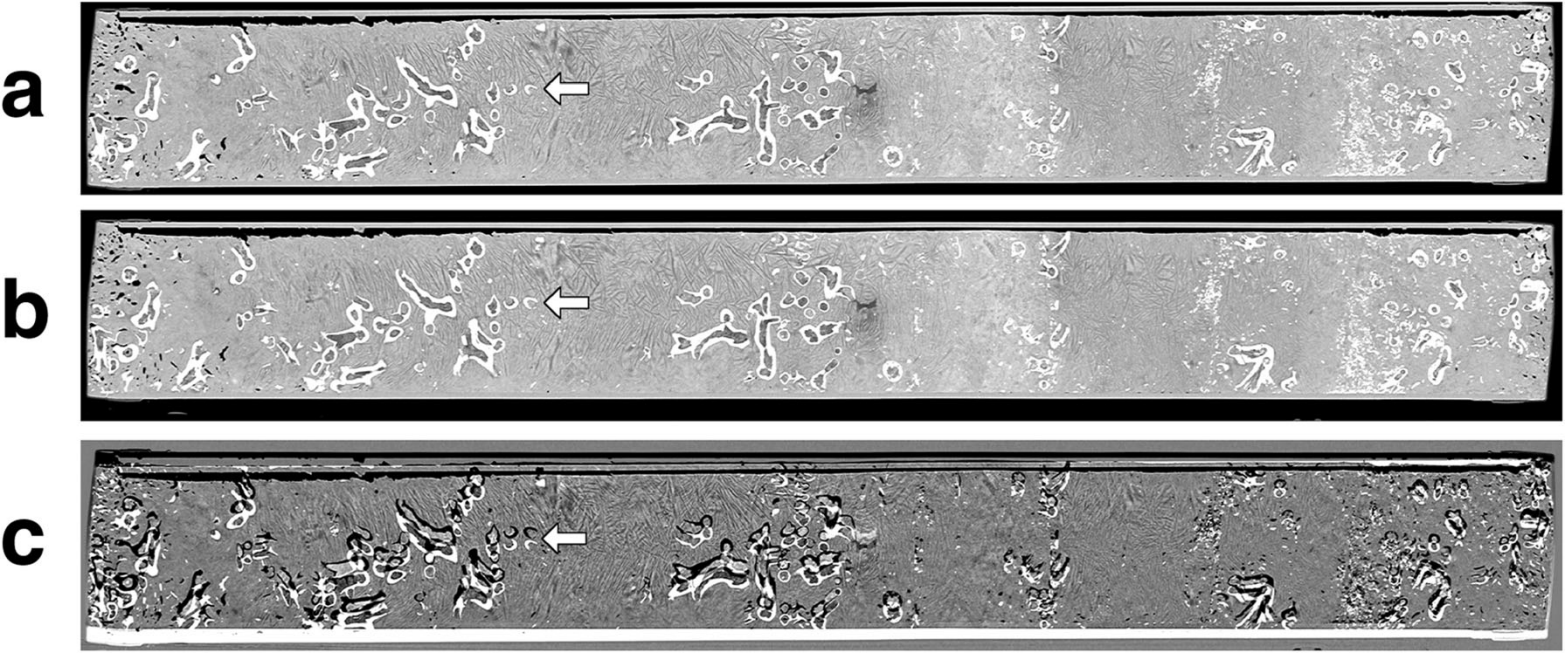
Examples of sagittal reconstructions of CT acquisitions of sediment cores, which were annotated with a digital ruler (not shown) and used during geological sampling to identify and locate coral fragments below the surface. **a** Before alignment correction. **b** After alignment correction. **c** Difference image before/after alignment correction. Areas with high density before correction and low density after correction are shown in white, areas with low density before correction and high density after correction are shown in black. Note the large differences in sample position in-plane and the smaller differences out-of-plane. For the effect of alignment correction on the sampling process, consider the coral fragment indicated by the arrow. As can be observed from the difference image, trying to sample this fragment at the location indicated by the uncorrected image would completely miss the fragment

Sagittal reconstructions with corrected alignment of sediment core sections with the CT scanner’s coordinate system could be calculated. Samples could successfully be taken from the core segments using CT images as guidance to identify coral fragments for minimally destructive sampling (Fig. 6).

## Discussion

The main goal of this study was the development of an alignment correction algorithm to facilitate the CT image-based analysis and systematic sampling of sediment cores. Based on the phantom analysis, the presented algorithm is able to restore alignment of scanned cylindrical objects with the main axes of the CT scanner’s coordinate system. While statistical analysis shows a significant difference between original and detected parameters, and the descriptive statistics show a relatively low minimum and high maximum (see Fig. 5), the highest average absolute difference in translation of 0.11 ± 0.08 mm was clearly below the height/width of a single voxel of 0.39 mm. For errors in rotation, the effect on the image will be largest at the end of the phantom and can consequently be estimated based on the average angle *α* = 0.15° and the phantom length *l* = 143 *mm* as follows:

2 $$ \Delta x=\tan \alpha \cdotp \frac{l}{2}=0.18\  mm $$

That is, the average absolute error based on the rotation of Δ*x* = 0.18 *mm* was clearly below the height/width of a single voxel of 0.39 mm. Still, differences in CT numbers measured in ROIs show the sensitivity of quantitative evaluations to misalignment (see Fig. 5). In general, parameters affecting alignment in the *x*-direction could be determined with a higher accuracy than parameters affecting alignment in the *y*-direction. This might be based on the fact that of the three points determined by the algorithm, two are established by line search in the *x*-direction.

By applying the alignment correction algorithm to the CT acquisitions of sediment cores containing CWCs, the alignment of the cores with the main axes of the CT scanner’s coordinate system could be restored. Sagittal reconstructions could be calculated, providing accurate image data as an aid for the sampling process of the cores. CWC fragments could successfully be extracted from the sediment cores for further analysis. In the future, further image-based analysis of, *e.g.*, CWC composition may be facilitated by the use of dual-energy CT.

Phantom alignment for analysis of standardised image quality phantoms in CT is often based on careful positioning of the phantom using the CT system’s laser sight and phantoms often provide markings to aid positioning [1]. Automatic post-processing and evaluation software for image quality phantoms may include some form of alignment correction as part of quantitative image analysis, which can be tailored to the individual phantoms, or manual correction may be performed on the acquired images [1, 7, 9]. Based on the results presented here, the algorithm could be applied to provide a generalised alignment correction for CT phantoms of various designs. Application of alignment correction could simplify phantom positioning before the acquisition as well as ROI placement and evaluation after acquisition, thus improving reproducibility of the analysis [10].

Some limitations of the presented algorithm and its evaluation have to be considered. Most importantly, the algorithm is unable to detect and/or correct rotations around the *z*-axis. This is a consequence of the fact that the CT images of the sediment cores do not offer any information on the rotation of the core. In future acquisitions, this could be alleviated by placing x-ray dense material (*e.g.*, a metal wire) along the demarcations for cutting at the end of the core, which would allow to determine rotation. For image quality phantoms, where the composition of the phantom is known *a priori*, a detection of defining features (*e.g*., low-contrast insets, wires for modulation-transfer-function determination) and their rotation relative to the CT coordinate system is possible [7, 9].

Furthermore, the algorithm is limited by the assumptions on the size and position of the object, depending on a successful line search to determine object position. However, the misalignment necessary to exceed these limits is so large that it would either be already apparent during the image acquisition process or result from operator errors during image reconstruction. The same reasoning can be applied to the choice of parameters used for phantom evaluation, which was limited in the magnitude of the parameters, which is a limitation resulting from the field of view of the original phantom acquisition. In practice, deviations larger than the ones applied here should easily be visible during the initial orientation of the phantom using the CT system’s laser sight.

In conclusion, the algorithm presented here was able to correct misalignment of scanned sediment cores, enabling the use of CT images to support core sampling for further analysis. Future application of the algorithm as part of the automated quantitative analysis of image quality phantoms seems to be possible.
